# Comparative analysis of actinorhizal nodule and associated soil microorganism diversity and structure in three *Alnus* species

**DOI:** 10.3389/fpls.2025.1572494

**Published:** 2025-05-08

**Authors:** Aizi Tong, Wei Liu, Xiaoliang Liu, Junyi Zhu, You Zhou, Jianhua Li

**Affiliations:** ^1^ Key Laboratory of Evaluation and Application of Changbai Mountain Biological Germplasm Resources of Jilin Province, College of Life Science, Tonghua Normal University, Tonghua, China; ^2^ Biology Department, Hope College, Holland, MI, United States

**Keywords:** *Alnus*, root nodules, 16S rRNA, *Frankia*, symbiosis

## Abstract

**Background:**

Due to the importance of biological nitrogen fixation in terrestrial ecosystems, actinorhizal symbiosis has attracted more and more attention. Alders (*Alnus*) are important actinorhizal plants, but little is known about the diversity of symbiotic microbiota in the actinorhizal nodules. In addition, it remains unclear about the influence of the host species and habitats on the microbial community of alder root nodules and rhizospheric soils.

**Methods:**

In this study we sequenced the hyper-variable regions of the 16S rRNA from the root nodules and their rhizosphere soils of three alder species (*Alnus mandshurica*, *A. sibirica*, *A. japonica*) in northeastern China to explore the diversity, composition, network association, and nitrogen cycling pathway of the microbial communities in the actinorhizal nodules and associated soils.

**Results:**

The results showed that the microbial community α-diversity decreased significantly from the associated soil to the root nodule, and the microbial diversity in the root nodule of *A. sibirica* was not affected by the habitats. The dominant microbe genus in alder nodules was *Frankia*, whose abundance was significantly higher than that in associated soil samples. Furthermore, the abundance of *Frankia* was affected by alder tree species, but not by the habitats. The most significant taxon in the nodules of all the three alders was *Frankia* genus, which was negatively correlated with other six genera of microbes. The main function of microorganisms in alder nodules is nitrogen fixation, which is not affected by tree species and their habitats.

**Conclusion:**

These findings suggest that the host determines the microbial community composition in the root nodule of three alders. This study provides valuable insights into the effects of alder species and habitats on the microbial communities of alder nodules and associated soils.

## 1 Introduction

Free nitrogen (N_2_) is abundant in the atmosphere, but it cannot be used directly by plants. Nitrogen acquisition by plants is mainly dependent on the biological nitrogen fixation, by which nitrogen-fixing microbes converting free nitrogen (N_2_) into the nitrogenous compound usable by plants. Therefore, biological nitrogen fixation plays an important role in soil nitrogen cycle (Roy et al., 2019; Soumare et al., 2020). The nitrogen-fixing microbe rhizobia live symbiotically with legumes and the actinorhizal *Frankia* form root nodules in eight woody plants families (Ardley and Sprent, 2021). The actinorhizal woody plant families (Rhamnaceae, Betulaceae, Datiscaceae, Coriariaceae, Casuarinaceae, Rosaceae, Myricaceae and Elaeagnaceae) contain 25 genera and more than 220 species (Benson and Dawson, 2008). *Frankia* not only improves plant growth, nitrogen content, and survival rate, but also alleviates abiotic and biotic stresses (Diagne et al., 2013).

Alders (*Alnus* sp.) is the only actinorhizal plant genus in Betulaceae with about 35 species and shows a worldwide geographic distribution (Normand et al., 2007). They are important nitrogen suppliers in the terrestrial ecosystem, and maintain ecological balance, thus have been used as important afforestation trees (Greer et al., 2008). *Frankia* strains fix nitrogen inside the root nodules (Gtari et al., 2013). *Frankia* strain isolation and cultivation were first reported in 1978 (Benson and Silvester, 1993; Callaham et al., 1978). However, the mechanism of nitrogen-fixing symbiosis has not been deeply studied due to the slow growth of *Frankia*, difficulty to isolate, easy contamination by other microbes in the culture process, and the great variation of strains in different hosts. Cloning and sequencing of the 16S rDNA have been successfully used to analyze the microbial diversity in the root nodules of various actinorhizal plants. It has been suggested that the *Frankia* diversity in the root nodule of actinorhizal plants may be correlated with host plant species and the geography (Balkan et al., 2019), but not with the abundance or relative diversity of *Frankia* populations in the rhizosphere soils (Tekaya et al., 2018). Despite these findings, the microbial community composition and nitrogen cycle of microbial function in the root nodules and associated soils of alders have not been sufficiently studied.

*Alnus mandshurica* (*Alnus mandshurica* (*Callier*) *Hand.-Mazz*.), *Alnus sibirica* (*Alnus sibirica Fisch*. ex *Turcz*.), and *Alnus japonica* (*Alnus japonica* (*Thunb*.) *Steud*.) are the three major tree species of *Alnus* genus in the northeast of China, and have been often used for soil and water retention and fire retardant barrier. Here we used the environmental 16S rRNA gene sequencing methods to identify microbial diversity in the root nodules and the rhizospheric soils and test the hypotheses that 1) the microbial communities are different greatly in the two places, and 2) the actinobacterial communities inside the root nodules are correlated with host species and bacterial diversities in the surrounding soils.

## 2 Materials and methods

### 2.1 Sample collection and processing

**Figure 1:**
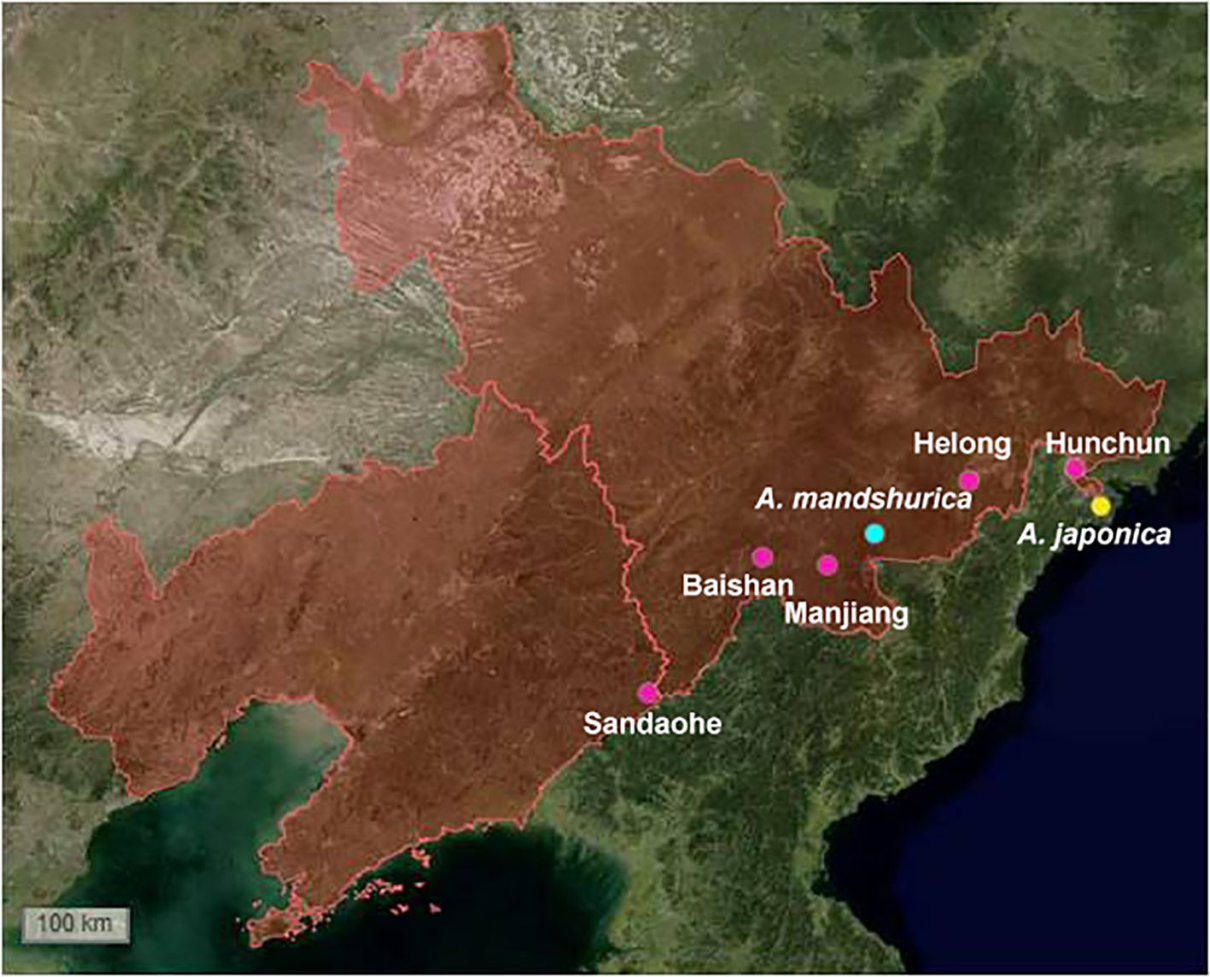
Sampling sites of *Alnus* species. The red zone includes Jilin and Liaoning provinces in northeast China. Green dot: *A. mandshurica*, Yellow dot: *A. japonica*, Pink dot: *A. sibirica*, and marked with place names in the map.

To explore the diversity of actinomycetes in alder root nodules and associated soils, we sampled root nodules and root nodule surface soils (associated soils or rhizosphere soils) of three alder species (*A. mandshurica*, *A. sibirica*, and *A. japonica*) in the northeast of China (**Figure 1**; **Supplementary Table S1**). Five plants were collected at each site and the plants were at least 20 meters apart to increase habitat and microbial diversity. Am_nodule, As_nodule, and Aj_nodule represent the root nodules of *A. mandshurica*, *A. sibirica*, *A. japonica*, and Am_soil, As_soil, and Aj_soil represent the associated soils of *A. mandshurica*, *A. sibirica*, *A. japonica*, respectively.

To ensure representative sampling, the three to five nodules collected from each plant were pooled as one nodular sample. The 1 mm thick soils layer attached to the nodule surface of each plant were collected as an associated soil sample. Five biological replicates were set for each nodule and soil sample. The fresh root nodules were disinfected with 70% (v/v) ethanol and washed three times in sterile distilled water.

### 2.2 DNA extraction, 16S amplicon generation, and sequencing

Total genomic DNAs were extracted from the soil samples using the E.Z.N.A.^®^ soil DNA Kit (Omega, U.S.) according to the manufacturer’s protocols. For root nodule samples, we used a combination of mechanical grinding and enzymatic lysis. Specifically, individual nodule samples were flash-frozen in liquid nitrogen and homogenized using a tissue homogenizer. The resulting powder was then subjected to enzymatic digestion with lysozyme (20 mg/mL, 37°C for 30 min) to enhance cell wall disruption. DNA from nodules was extracted using the DNeasy PowerLyzer PowerSoil Kit (QIAGEN, Cat No. 12855) according to operating instruction. The concentration and purification of the extracted DNA were first determined by the NanoDrop 2000 ultraviolet-visible spectrophotometer (Thermo Scientific, U.S.), and the quality was evaluated by 1.0% agarose gel electrophoresis. The V3-V4 hyper-variable regions of the bacterial 16S rRNA gene were amplified using forward primer 338F (5’- ACTCCTACGGGAGGCAGCAG-3’) and reverse primer 806R (5’-GGACTACHVGGGTWTCTAAT-3’) (Xu et al., 2016) in the GeneAmp^®^ 9700 thermocycler (ABI, USA). The PCR reactions were conducted using the following program: 3 min of denaturation at 95°C; 27 cycles of denature at 95°C for 30 s, anneal at 55°C for 30s, and elongation at 72°C for 45 s; and a final extension at 72°C for 10 min after the cycle. The PCR reactions system were performed in triplicate 20 μL mixture containing 4 μL of 5 × FastPfu Buffer, 2 μL of 2.5 mM dNTPs, 0.8 μL of each primer (5 μM), 0.4 μL of FastPfu Polymerase and 10 ng of template DNA. The PCR fragments were extracted from 2.0% agarose gel and further purified using the AxyPrep DNA Gel Extraction Kit (Axygen Biosciences, USA) and quantified using QuantiFluor™-ST (Promega, USA). Purified amplicons were pooled in equimolar ratio and paired-end sequenced (2 × 300) on the Illumina MiSeq platform (TruSeq™ DNA SamplePrep Kit, Illumina, U.S.) following the standard protocols by Majorbio Bio-Pharm Technology Co. Ltd. (Shanghai, China). The raw reads were deposited the NCBI Sequence Read Archive (SRA) database (Accession Number: PRJNA1206778).

### 2.3 Sequence data processing

Raw fastQ files were demultiplexed and then quality-filtered by fastp (https://github.com/OpenGene/fastp, version 0.19.6) and merged by FLASH (http://www.cbcb.umd.edu/software/flash, version 1.2.11) with the following criteria: (i) The reads were truncated at any site receiving an average quality score < 20 over a 50 bp sliding window. (ii) Primers were exactly matched allowing 2 nucleotide mismatches, and reads containing ambiguous bases were removed. (iii) Sequences whose overlap longer than 10 bp were merged according to their overlap sequence.

The UPARSE v7.1 (http://drive5.com/uparse/) software was used to cluster operational taxonomic units (OTUs) based on 97% similarity cut off, and the UCHIME (version 4.2) was used to identify and remove the chimeric sequences. The sequences of each sample were compared with the Silva bacterial 16S rRNA database (SSU132) by RDP classifier (http://rdp.cme.msu.edu/, version 2.11) using a confidence threshold of 70%. Then, the data were analyzed using the online Majorbio I-Sanger Cloud Platform (www.i-sanger.com).

### 2.4 Statistical analyses

Mothur software (version 1.30.2) was used to analyze the α-diversity index of microbial communities, and the Student’s t-test and one-way analysis of variance (ANOVA) were used to compare the α-diversity index between two different groups and among multiple groups, respectively. For the β-diversity, the Principal Coordinates Analysis (PCoA) was used to calculate the distance between samples base on the Bray-Curtis distance algorithm, and the PERMANOVA test was employed to analyze the impact of different grouping factors on sample differences. The Welch T test and one-way ANOVA were used to determine whether significant differences existed in microbial abundance. The LEfSe (Linear discriminant analysis Effect Size; http://huttenhower.sph.harvard.edu/LEfSe) analysis was used to identify the bacterial taxon with significant difference in abundance (the LDA score > 4.0; *p* < 0.05) from phylum to species levels among the different groups. The correlation networks of top 30 genera were constructed to explore the internal community relationships across the groups based on spearman (|r| > 0.6; *p* < 0.05). The FAPROTAX dataset was used to predict the function of the microbial community (Liu et al., 2023), and the Welch T test and one-way ANOVA were employed to determine whether significant differences existed in the abundance of microbial functions. A *p*-value less than 0.05 were considered statistically significant (*p < 0.05, **p < 0.01, and ***p < 0.001).

## 3 Results

### 3.1 Microbial community diversity in root nodules and associated soils

Out of the 2594271 valid reads, we obtained the microbial 16S rRNA genes, with an average sequence length of 433 bp (**Supplementary Table S1**). By comparison with the genes in the Silva database, we identified the bacterial communities to 40 phyla, 100 classes, 187 orders, 366 families, 671 genera, and 1461 species of microorganisms in the root nodules and associated soils of the three alder species. We clustered high-quality sequences with a similarity of 97% into 4407 bacterial operational taxonomic units (OTUs) (**Supplementary Table S4**). Significant differences in the OTUs were found between the root nodules and associated soils of the three alder species. The number of OTUs in the associated soils was higher than that in the root nodules, and the numbers of OTUs in As_bs (soil) was the highest (2588) and 3.1 times that of the lowest (Am_nodule: 843). There were differences in the numbers of specific and shared OTUs in different groups. The numbers of shared OTUs in soil samples (707, 1150) were significantly higher than these in root nodules (235, 503) (**Supplementary Figure S1**). It is worth noting that the root nodule sterilization cannot fully confirm that soil microbial DNA has not entered the nodule DNA sample, so some carryover of soil microbiota is possible.

**Figure 2:**
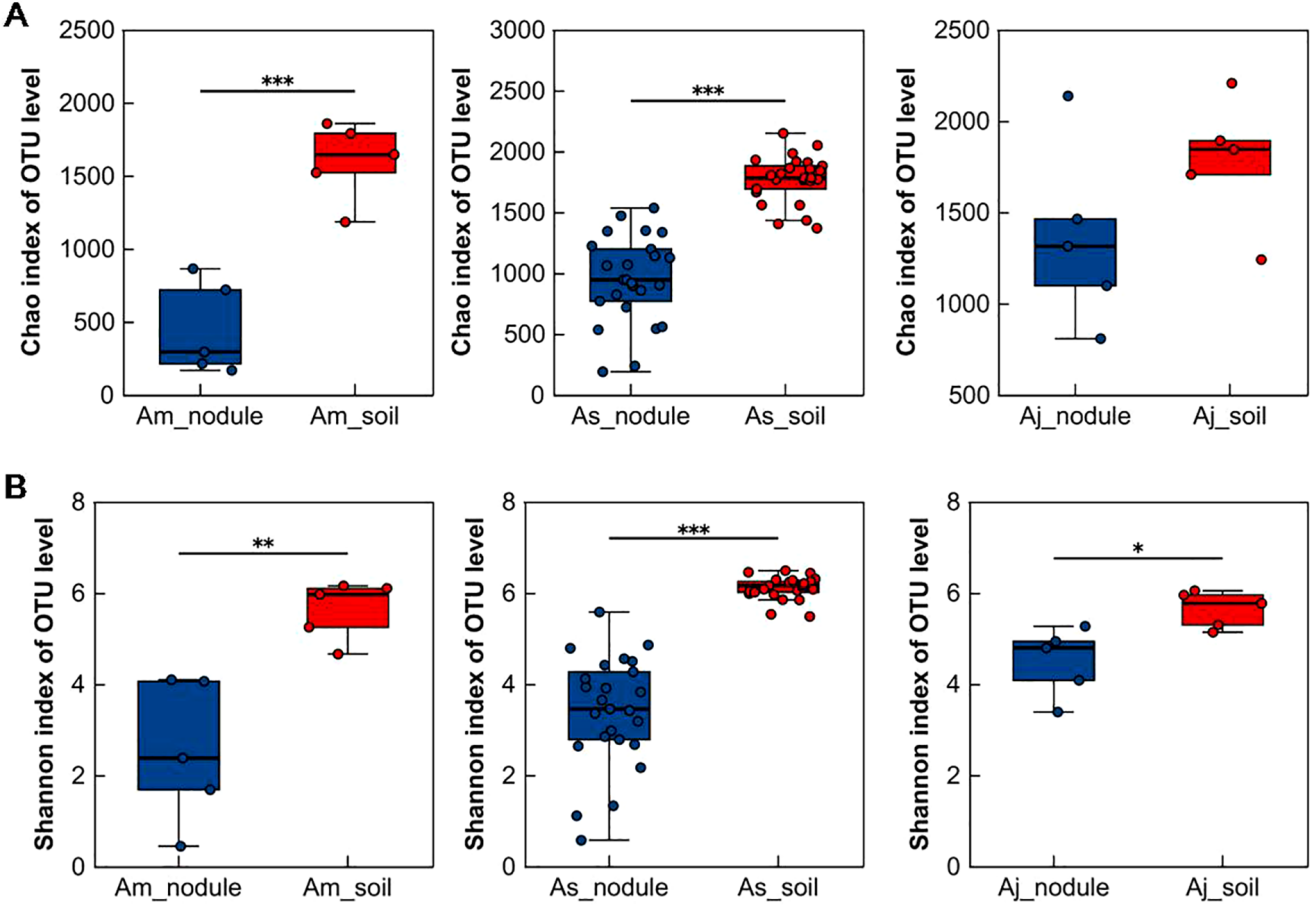
Microbial community α-diversity in root nodules and associated soils of different alders. **(A)** Comparison of microbial community Chao index between root nodules of three different alder species and associated soils. **(B)** Comparison of microbial community Shannon index between root nodules of three different alder species and associated soils. The Student’s t-test was used to compare the two groups. Am_nodule, As_nodule, and Aj_nodule represent the root nodules of *A mandshurica*, *A sibirica*, *A japonica*, and Am_soil, As_soil, and Aj_soil represent the associated soils of *A mandshurica*, *A sibirica*, *A japonica*, respectively. The same applies below. Asterisks indicate significant differences between samples, *p < 0.05, **p < 0.01, ***p < 0.001.

We calculated the Chao and Shannon indices based on 16S amplicon data to determine the distribution characteristics of microbial diversity in two different sample types (root nodules and associated soils) (Deng et al., 2024). The Chao index in Am_soil and As_soil was significantly higher than that in Am_nodule and As_nodule, indicating that the richness of Am_soil and As_soil was higher than that of Am_nodule and As_nodule, respectively (**Figure 2A**). The richness between Aj_nodule and Aj_soil was not significantly different. The Shannon indices of Am_soil, As_soil, and Aj_soil were significantly higher than those of Am_nodule, As_nodule, and Aj_nodule, indicating that the microbial community α-diversity in associated soils was higher than that in the root nodules (**Figure 2B**). In addition, there were differences in the α-diversity of the microbial community in root nodules and soil samples of the three different alder species by comparing the Chao and Shannon indices (**Supplementary Figures S2A, C**). However, there were no significant differences in the α-diversity of the microbial community in the root nodules and associated soils sampled from five different habitats of *A. sibirica*, suggesting that there was no association between microbial community α-diversity in the root nodules and associated soils of *A. sibirica* and changes in growing environments (**Supplementary Figures S2B, D**).

**Figure 3:**
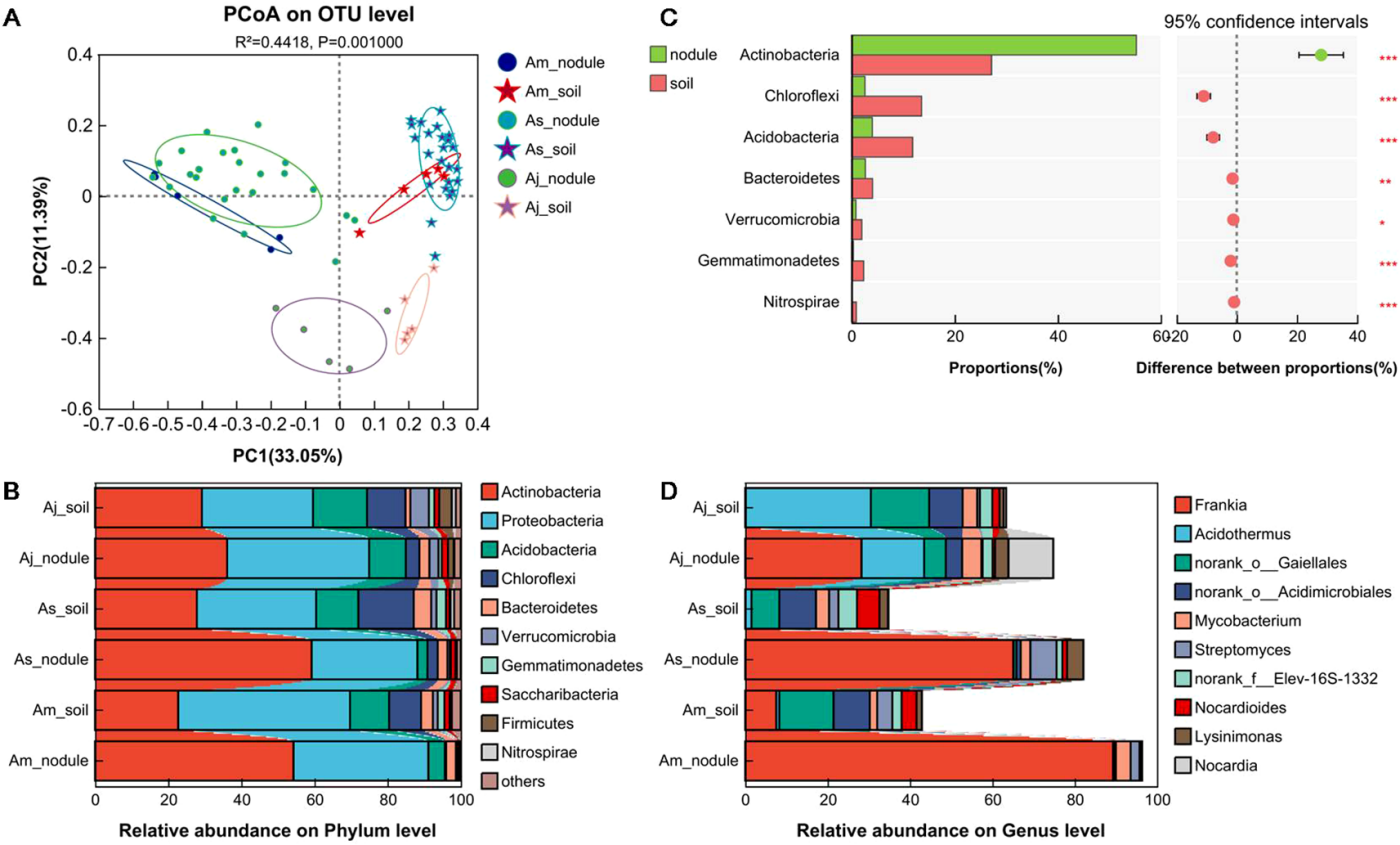
Microbial community β-diversity and constituent structure in root nodules and associated soils of three alder species. **(A)** PCoA analysis of six different groups on OTU level. **(B)** Relative abundance of the top ten dominant phyla in microbial community of six different groups. **(C)** Difference analysis of the abundance of ten dominant phyla between nodule and soil. **(D)** Relative abundance of the top ten dominant genera in Actinobacteria phylum of microbial community. PERMANOVA was used to analyze the impact of different grouping factors on sample differences. Only differentially abundant phyla were shown in Figure **(B)** The Welch T test was used to compare the two groups. The nodule represent the root nodules of different alders, soil represent the associated soils of three alders, respectively. Asterisks indicate significant differences between samples, *p < 0.05, **p < 0.01, ***p < 0.001.

The Bray-Curtis distance, PCoA and PERMANOVA analyses showed that sample types had a significant effect on β-diversity of microbial communities (*p* = 0.001; **Figure 3A**). The sample types (nodule and soil) alone explained 33.05% of the total variation in the microbial community composition (PERMANOVA, *p* = 0.001). Subsequently, the root nodule and associated soil samples of three different alder species were analyzed, respectively. For the total variation of microbial community composition, the root nodules explained 34.09% (PERMANOVA, *p* = 0.001) and associated soils explained 19.42% (PERMANOVA, *p* = 0.001) (**Supplementary Figures S3A, C**). However, for *A. sibirica* from different sites, there was no significant difference in the microbial community composition of the root nodules, but considerable difference in the associated soil samples (root nodule, *p* = 0.07; associated soils, *p* = 0.001; **Supplementary Figures S3B, D**). These results indicated that the microbial community composition in the root nodule of *A. sibirica* was not affected by the habitats.

### 3.2 Specific differences in microbiome between root nodules and associated soils

In the three alder species, the microbiota in the root nodule and rhizospheric soil samples belonged to 40 phyla (only shown the top ten dominant phyla, **Figure 3B**). However, the abundance differed greatly. In the root nodules, Actinobacteria (36.12~59.22%) and Proteobacteria (28.96~38.84%) were most abundant. In contrast, the microbiota consisted of Proteobacteria (30.39~47.00%), Actinobacteria (22.74~29.20%), Chloroflexi (8.72~15.16%) and Acidobacteria (10.71~14.80%, **Figure 3B**) in the soil. Chloroflexi, Acidobacteria, Bacteroidetes, Verrucomicrobia, Gemmatimonadetes, and Nitrospirae were significantly less abundant in the nodules than soils (Welch T test, *p* < 0.05, bonferroni-corrected, **Figure 3C**). The abundance of the top ten dominant phyla in root nodules and soils differed significantly in different alders. In the root nodules, the abundance of six phyla (Actinobacteria, Proteobacteria, Acidobacteria, Chloroflexi, Saccharibacteria, and Gemmatimonadetes) was significantly different, while Bacteroidetes, Gemmatimonadetes, and Nitrospirae showed differences in soil samples (**Supplementary Figures S4A, B**). There was no significant difference in the abundance of the 10 dominant phyla in the root nodules of *A. sibirica* collected from five differed sites, but Actinobacteria and Gemmatimonadetes showed remarkable difference in soil samples (**Supplementary Figures S4C, D**). This indicated that the microbial community compositions in the root nodules of *A. sibirica* were not affected by habitats. Although there was difference in the abundance of Actinobacteria in the different soils, there was no difference in the alder nodules and the abundance in nodules higher than in soils, indicating that the root nodules of *A. sibirica* promoted the enrichment of Actinobacteria.

At the genus level, *Frankia* was the most abundant Actinobacteria in the nodules of the three species (*A. mandshurica*: 89.29%, *A. sibirica*: 65.07%, *A. japonica*: 28.24%), but showed a much lower proportion in the associated soils (7.53%, 0.09%, and 0.14%) (**Figure 3D**). The abundance of *Frankia* in the root nodules of the three alder species was significantly higher than that in the associated soils (Welch T test, *p* < 0.05, bonferroni-corrected, **Supplementary Figure S5A**). The abundance of *Frankia* in the nodules was significantly different among the alder species, but similar in the associated soils (one-way ANOVA test, *p* < 0.05, **Supplementary Figure S5B**). Moreover, there was no difference in the abundance of *Frankia* in the nodule and soil samples of *A. sibirica* from five different habitats (one-way ANOVA test, *p* > 0.05, **Supplementary Figure S5C**). The dominant genus of Actinobacteria in the root nodules of alders was *Frankia*, and its abundance was significantly higher than that in the associated soils. The abundance of *Frankia* in nodules was affected by alder tree species, but not by it habitats.

### 3.3 Difference analysis of microorganism in intergroup samples

**Figure 4:**
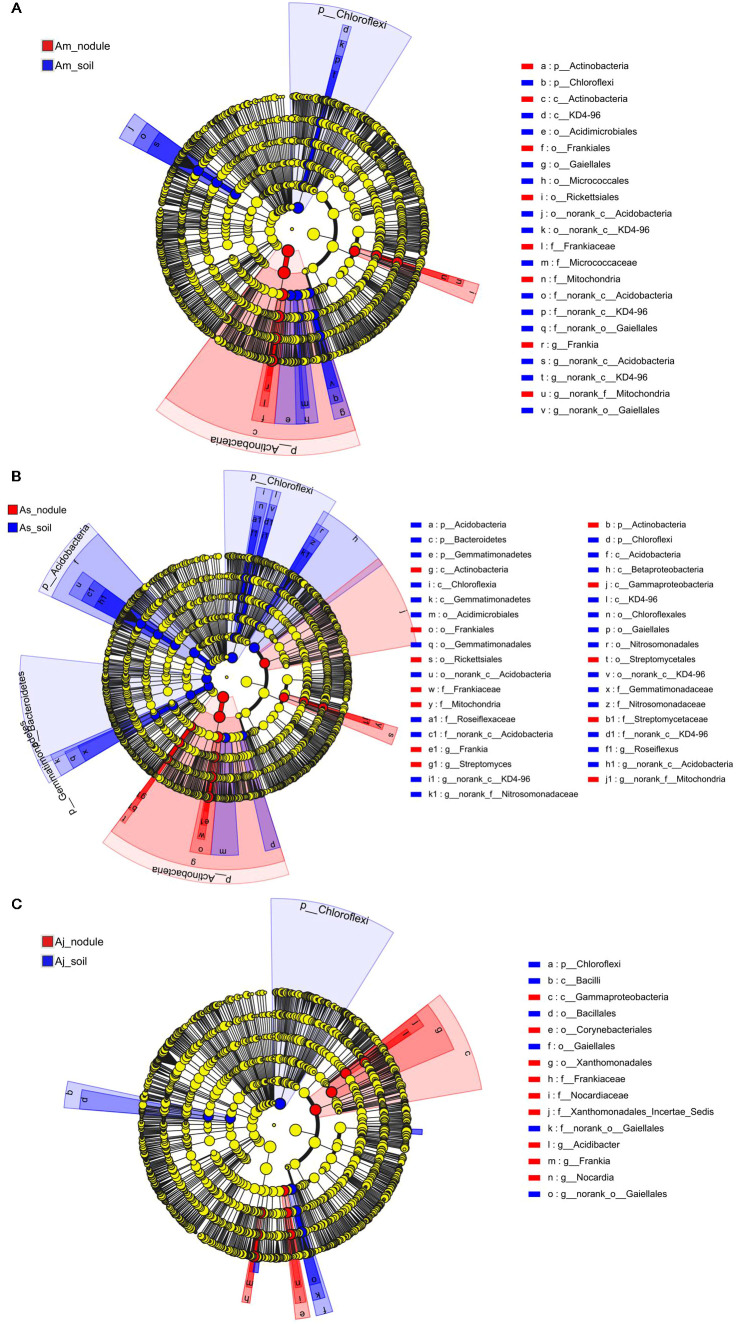
LDA effect size (LEfSe) analysis of the *A mandshurica* **(A)**, *A sibirica* **(B)**, and *A japonica* **(C)** microbial communities with an LDA score of > 4.0 (*p* < 0.05). Phylogenetic levels from phylum to species are represented by circles. Different-colored nodes represent microbial groups that are significantly enriched in the corresponding groups and have a significant influence on the differences between groups. The yellow nodes represent microbial groups that have no significant difference between groups or have no significant effect on the differences between groups.

The LEfSe analysis was used to identify the dominant taxon between the root nodules and the associated soils of three distinct alder species (LDA score > 4.0, *p* < 0.05, **Figure 4**). The microorganism with high relative abundances among nodules and soil samples of different alders exhibited remarkable differences. *Frankia* was the biomarker taxon of all root nodule samples in three alders. Additionally, the *Rickettsial*es was dominant in nodules of *A. mandshurica* (**Figure 4A**). Beside the *Rickettsiales*, *Gammaproteobacteria* and *Streptomyces* were dominant in the nodules of *A. sibirica* (**Figure 4B**). *A. japonica* nodules were clearly different from nodules of *A. mandshurica* and *A. sibirica*, and the dominant taxa of *A. japonica* were *Nocardia* and *Acidibacter* (**Figure 4C**). Moreover, the class of Acidobacteria and KD4-96, *Acidimicrobiales*, *Micrococcaceae*, and Gaiellales order were dominant taxa for associated soils in *A. mandshurica* (**Figure 4A**). The class of Acidobacteria and KD4-96, *Acidimicrobiales*, *Roseiflexus*, Nitrosomonadaceae family, *Gaiellales*, *Gemmatimonadaceae*, and Bacteroidetes were dominant taxa for associated soils in *A. sibirica* (**Figure 4B**). Chloroflexi, uncultured Actinoallomurus sp., Gaiellales, uncultured bacterium of the family Xanthobacteraceae, and *Bacillales* were dominant taxa for the soils of *A. japonica* (**Figure 4C**).

The LDA histogram showed that the soils (24 taxa) had more remarkable taxa than nodules (10 taxa), and the genus *Frankia* was the most significant biomarker taxon in the root nodules (LDA score > 4.0, *p* < 0.05, **Supplementary Figure S6A**), thus suggesting that the distinction between microbial communities of nodules and associated soils was mainly determined by *Frankia*. Moreover, the histogram of the LDA value distribution revealed that 10 taxa were enriched including three genus (*Frankia*, *Burkholderia-Paraburkholderia*, and *Granulicella*) in the Am_nodule, 7 taxa were enriched including one genera (*Streptomyces*) in the As_nodule, and 23 taxa were enriched including five taxa (*Acidothermus*, *Nocardia*, *Acidibacter*, Acidobacteria class, and Acidobacteriaceae family Subgroup 1) in the Aj_nodule, respectively (**Supplementary Figure S6B**). In the three group soil samples, five dominant taxa were found in the samples of Am_soil, seven dominant taxa were identified in the samples of As_soil, while twenty dominant taxa were found in the samples of Aj_soil (**Supplementary Figure S6C**). In the microbial taxon categories of the Am_nodule, *Frankia* genus with an LDA score of > 5.0 was extremely significant, signifying that the microbial composition in the nodules of *A. mandshurica* was different from the microbial composition in the nodules of *A. sibirica* and *A. japonica* (**Supplementary Figure S6B**). Furthermore, the most significant microbial taxon in the Am_soil was *Betaproteobacteria* class (LDA score of = 4.76). Studies have also shown that the communities of the nodules and soils of *A. japonica* had more dominant taxa than the nodules and soils of *A. mandshurica* and *A. sibirica* (**Supplementary Figures S6B, C**).

### 3.4 *Frankia* in root nodules was negatively correlated with the other genera

**Figure 5:**
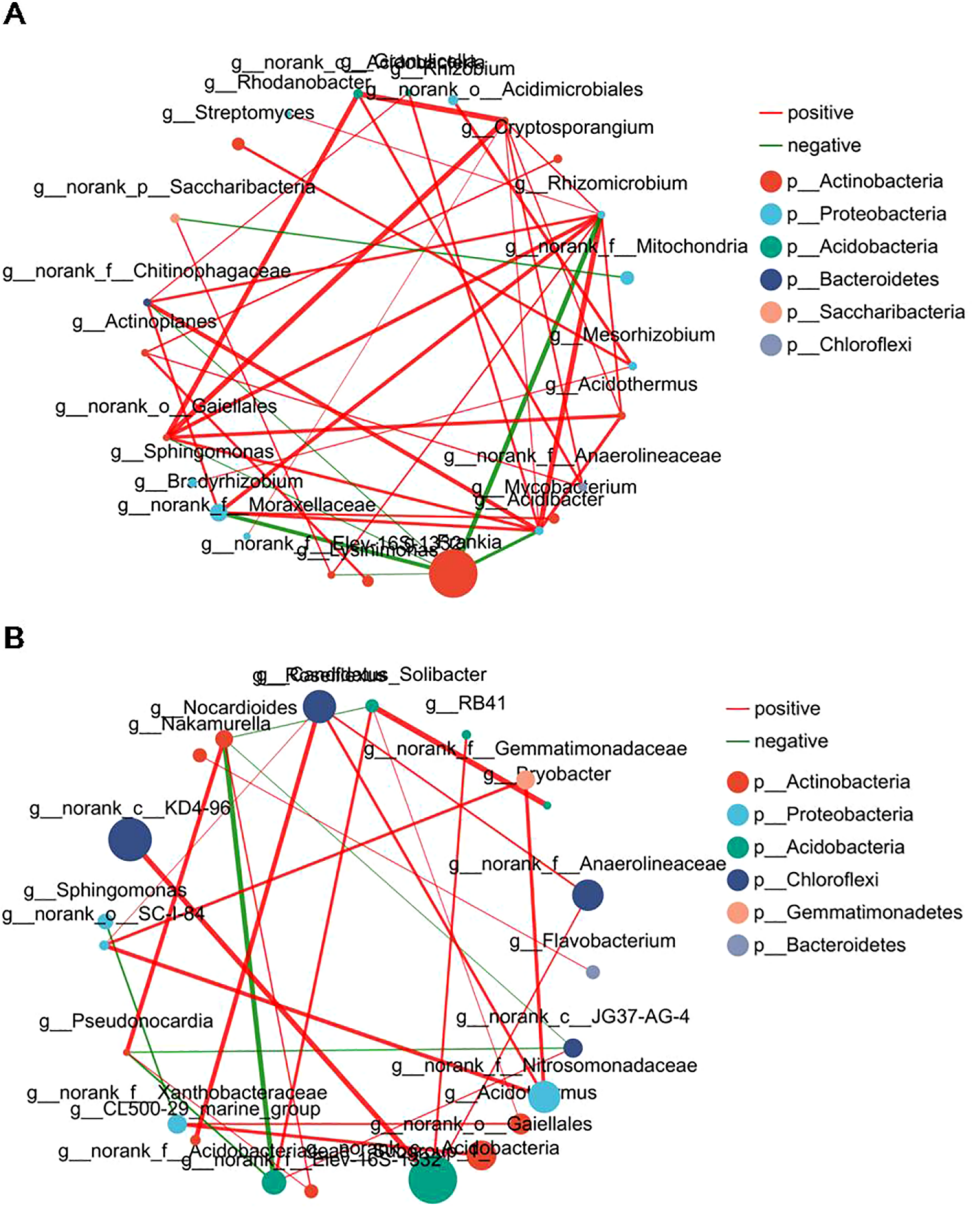
Interaction network of dominant microbiota at the genus level (top 30) in root nodules and associated soils. **(A)** Interaction network in root nodule. **(B)** Interaction network in root nodule surface soils. The size of the nodes shows the abundance of the genus, and the different colors indicate the corresponding taxonomic assignment at the phylum level. The edge color represents positive (red) and negative (green) correlations. The edge thickness indicates the correlation values; only show significant interactions are shown (|r| > 0.6; *p* < 0.05).

Symbiotic networks are composed of various interspecific interactions in which species can be used to investigate correlations among microbial groups (Banerjee et al., 2018). To understand the correlation and complexity of the microbial community in the root nodules and associated soils of three alders, symbiotic network analyses were used at the top 30 genera. Our analysis results revealed 39 and 26 correlations among the genera in the root nodules (**Figure 5A**; **Supplementary Table S2**) and the associated soils (**Figure 5B**; **Supplementary Table S3**), respectively. In general, the nodule networks were more linked and complicated than the soil networks. The symbiotic networks of root nodules and associated soils both contain 6 distinct phyla, among which root nodules of alder had a higher proportion of nodes belonging to Actinobacteria (10 genera). The dominant *Frankia* in the nodules was negatively correlated with six genera, especially *Rhizomicrobium* (-0.81), *Bradyrhizobium* (-0.78), and *Acidibacter* (-0.74), speculating that this may be due to the symbiotic relationship between alders and *Frankia*, resulting in the enrichment of *Frankia* in the nodules and hinder the colonization of other microorganisms in the nodule of alders.

### 3.5 Functional prediction of microbial communities of root nodules and associated soils

**Figure 6:**
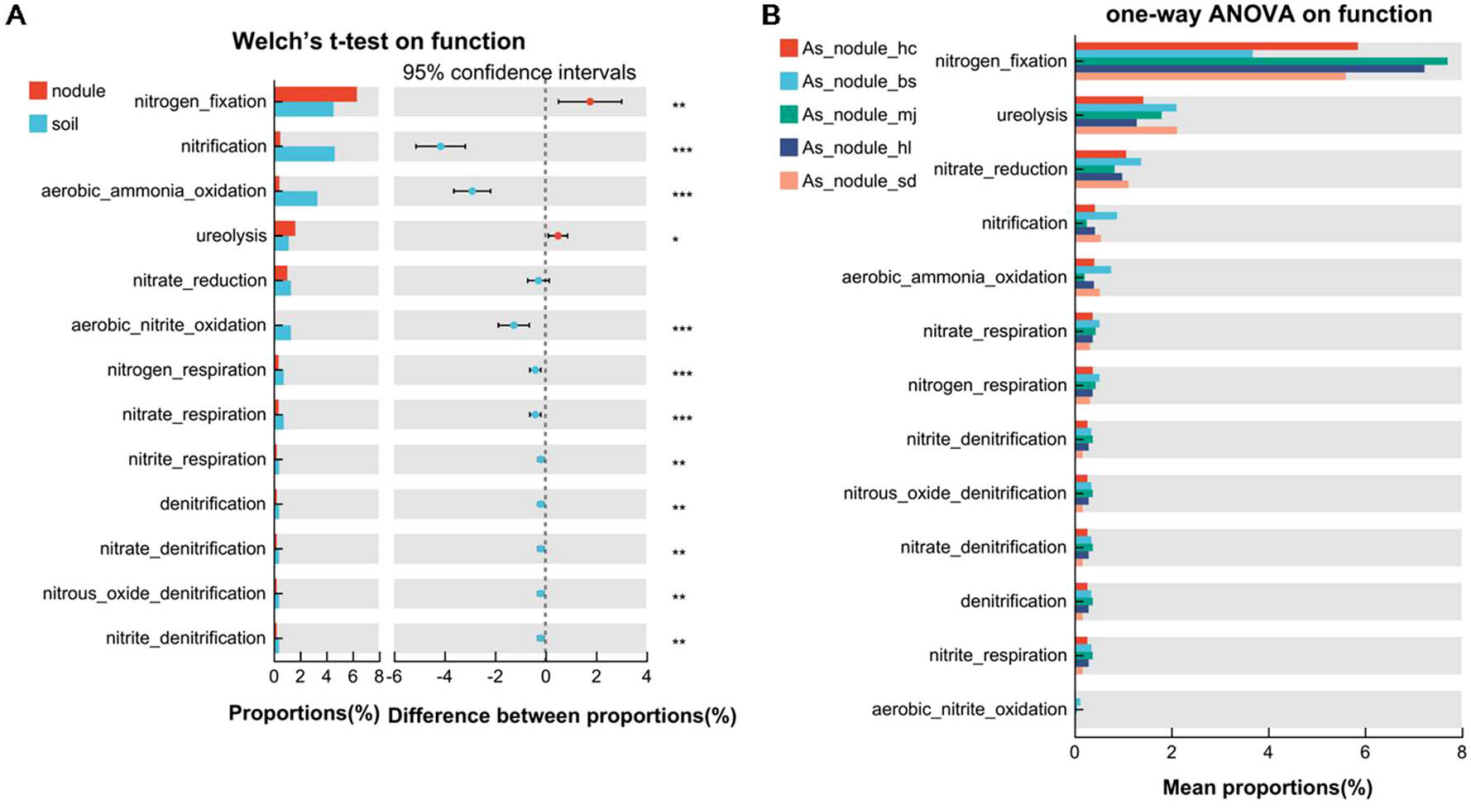
Comparison analysis of the abundance of the microbial nitrogen cycling pathways among different nodule and soil samples. **(A)** The differential abundance of the microbial nitrogen cycling pathways between nodules and soils. **(B)** The differential abundance of the microbial nitrogen cycling pathways in root nodules of *A sibirica* from five different eco-geographical environments. The Welch T test and one-way ANOVA were used to compare multiple groups. Asterisks indicate significant differences among groups, *p < 0.05, **p < 0.01, ***p < 0.001.

The functional prediction of the microbial community using the FAPROTAX dataset recognized microbial functions related to nitrogen cycle pathways and the top functions included nitrogen fixation, nitrification, aerobic ammonia oxidation, ureolysis, nitrate reduction, aerobic nitrite oxidation, nitrate respiration, nitrogen respiration, nitrite respiration, nitrate denitrification, nitrite denitrification, nitrous oxide denitrification, and denitrification. In general, nitrogen fixation (37.80%) was the most common microbial nitrogen cycle pathway, followed by nitrification (13.74%), aerobic ammonia oxidation (10.15%), ureolysis (9.74%), and nitrate reduction (7.64%). The abundance of the other eight microbial functions was low. Significant differences were found in the functions of microbial communities between the root nodules and soils of the alders (**Figure 6A**). In particular, nitrogen fixation and ureolysis were significantly higher in the root nodules than in the associated soil (Welch T test, *p* < 0.05). Reversely, nitrification and aerobic ammonia oxidation pathways were significantly lower in the nodules than in the associated soils. There was no significant differences in all of the 13 nitrogen cycle pathways in the root nodules of *A. sibirica* populations (**Figure 6B**), while there were significant differences in associated soils among the sites of *A. sibirica* in nitrification, aerobic ammonia oxidation, ureolysis, nitrate_respiration, and nitrogen_respiration (**Supplementary Figure S7A**). These results illustrates that the nitrogen cycling pathways were not susceptible to environmental influences in the stable environment of the root nodules. In addition, the nitrogen fixation function was most abundant in the 13 nitrogen cycle pathways, and the abundance of nitrogen fixation function was not significant different in the root nodules among the three alder species (**Supplementary Figure S7B**). This assumes that different alder species have similar nitrogen fixation ability. Except for nitrification and aerobic nitrite oxidation, the other 11 nitrogen cycling pathways showed significant differences in the nodule superficial soils of three alders, indicating that the nitrogen cycling function in the soils was unstable in different alder species (**Supplementary Figure S7C**).

## 4 Discussion

### 4.1 The microbiota of root nodules and soil differ significantly

The OTUs number and α-diversity of microorganisms in root nodules were significantly lower than those in the associated soils (**Figure 2**; **Supplementary Figure S1**), which is consistent with the results in soybean and sea buckthorn (*Hippophae* L.) nodules (Han et al., 2020; Liu et al., 2024). This differential trend was consistent across the three different alder species, possibly because only a small fraction of the soil microbes entered the root system via interactions with the host and selective filtration of host. This may be due to the interaction between plants and soil microbes, and the plants provide some nutrients to the soil microorganisms for their growth and reproduction. Plants recruit root-related microbiota from the soil microbiome, so the density of microbes in the root endosphere was lower than that in the rhizosphere (Yamamoto et al., 2018). The microbial community composition of root nodules was different from that of the associated soil (**Figure 3A**). Some studies found that the rhizosphere and plant endosphere microbial communities were primarily driven by soil and host, respectively (Brown et al., 2020; Xiao et al., 2017). The microbial community composition in endophytic region of plants depends on host variety, genotype and ecological niches (Xiong et al., 2021). For example, plant species and genotypes influence the composition and abundance of rhizobia in legume nodules (Xiao et al., 2017). We discovered that the microbial community compositions were significantly different at the phylum and other taxonomic levels between nodules and soil (**Figure 3C**). The root nodules and associated soils were dominated by Actinobacteria and Proteobacteria, respectively. The differences may be related to biogeographic patterns and assembly process of the microbial communities (Li et al., 2022).

The compositions of endophytic bacterial community maybe mainly affected by the species, function and tissue of the host (Laforest-Lapointe et al., 2017; Wearn et al., 2012; Yuan et al., 2023). We found that the abundances of six (Actinobacteria, Proteobacteria, Acidobacteria, Chloroflexi, Saccharibacteria, and Gemmatimonadetes) of the ten dominant phyla in the nodules were significantly different among the host species (**Supplementary Figure S4A**). The result is consistent with the conclusion of comparative analysis of microbial community composition and structure in the root nodules of other alder species (Põlme et al., 2014; Vemulapally et al., 2022; Yuan et al., 2023). Host plants have also been shown to affect the microbial communities in maize (Zea mays), and wheat (Triticum aestivum)/barley (Hordeum vulgare) (Xiong et al., 2021).

The abundance of Actinobacteria in root nodules was significantly higher than that in the associated soils (Welch T test, *p* < 0.05, bonferroni-corrected, **Figure 3C**), indicating that the enrichment of Actinobacteria in the nodules. In addition, the abundance of Actinobacteria in the root nodules was mainly dominated by *Frankia*, which is significantly higher in the root nodules than in the soils, supporting the results from Vemulapally et al. (2022). These conclusions suggest that rhizosphere soils have a high abundance and diversity microbial communities with multiple functions that play key roles in the ecosystem, in contrast to the highly specific and relatively stable structural characteristics of microbial communities in root nodules (Sneha et al., 2021; Xu et al., 2023).

### 4.2 The microbial community in root nodules is not affected by local habitats

The α- and β- diversities of microbial communities in root nodules of *A. sibirica* from five different habitats were not significantly different (*p* > 0.05; **Supplementary Figures S2D, S3B**), and the abundances of all the 10 dominant phyla were not significantly different in nodule samples (**Supplementary Figure S4D**). These indicated that the microbial diversity and community composition structure of the root nodules was not affected by the habitats. Despite the large geographical range of *A. sibirica*, the microbial community in the root nodules remained relatively stable, suggesting that the microorganisms of the alder nodules have an intrinsic stability mechanism. The results we obtained are different from the conclusions of previous studies on nodular microorganisms. For example, the nitrogen-fixing microbes in legume nodules are significantly influenced by environmental factors, exhibiting a strong biogeographical pattern (Han et al., 2009; Zhang et al., 2017). The composition and abundance of rhizobia in legume nodules were influenced by soil physicochemical characteristics (Stefan et al., 2018) and geographical environment characteristics (Zhang et al., 2018). *Frankia* in the nodules of *Casuarina glauca* is influenced by environmental factors, with being less abundant under more arid environments (Ghodhbane-Gtari et al., 2021). Soil environmental conditions have a role in the selection of *Frankia* strains for root nodule formation of 12 *Alnus* taxa (Pokharel et al., 2011). Small-scale spatial variation and microenvironment conditions can affect the host-specificity of *Frankia* communities in red alder (*A. rubra*) and Sitka alder (*A. viridis*) (Wolfe et al., 2022).

### 4.3 Nitrogen fixation is the most dominant nitrogen cycle pathways in root nodules, and there is no difference of tree species

Previous studies have found that microorganisms selectively enriched in the rhizosphere and endosphere of plants may be associated with specific functions. Our study discovered selective enrichment of *Frankia* in the root nodules, and these microbes may have important functions in the alders. Nitrogen is a limiting factor for plant growth and development, which is mainly acquired through microbial mediated nitrogen fixation process by plants (Kuypers et al., 2018). Nitrogen fixation is the predominant nitrogen cycling pathway in microbial community of alder root nodules, and the proportion of nitrogen fixation in alder nodules was significantly higher than that in associated soils (Welch T test, *p* < 0.05, **Figure 6A**). The functional connection between bacteria and hosts has been documented in other plant partial systems (Brown et al., 2020; Pathan et al., 2018; Wang et al., 2021). In addition to *Frankia*, we also found non-*Frankia* microorganisms in alder root nodules (**Figure 3D**) that have promote plant growth and induce an earlier onset of nodulation (Ghodhbane-Gtari et al., 2019). However, there were no significant differences in the 13 nitrogen cycling pathways of nodules in *A. sibirica* collected from five different habitats. Therefore, the nitrogen cycling pathways may not be susceptible to the growing environment influences in the stable root nodule environment (**Figure 6B**). The abundance of nitrogen fixation in root nodules did not differ significantly among the three alder species (**Supplementary Figure S7B**). Moreover, our analysis also discovered that the proportion of nitrate reduction in the root nodules was high (**Figure 6B**). The results suggest that the evolution of alder trees and actinobacteria has produced a robust system that benefits both organisms in various environments (Liu et al., 2024).

## 5 Conclusions

We explored the microbiota in the root nodules and the associated soils by sequencing 16S rRNA genes. The root nodules show significantly less microbial diversity than the rhizospheric soils, indicating the selective filtering effect on nodule microbes. The abundance of *Frankia* may not have been affected by changes in the eco-geographical sites, indicating that the specificity and stability of symbiotic nitrogen fixation among alders. We have observed significant differences in the nitrogen cycle pathways of microbial communities between root nodules and associated soils. Nitrogen fixation abilities of the root nodules in three alders are not different, but greater than those of associated soils. The soil microbial community is mainly dominated by nitrogen fixation and nitrification, while the root nodule microbial community is mainly dominated by nitrogen fixation.

## Data Availability

The datasets presented in this study can be found in online repositories. The names of the repository/repositories and accession number(s) can be found below: https://www.ncbi.nlm.nih.gov/, Accession Number: PRJNA1206778.
