# Supplementary material for: Comparative analysis of actinorhizal nodule and associated soil microorganism diversity and structure in three Alnus species
Source: Front Plant Sci. 2025 May 8;16:1572494. doi: 10.3389/fpls.2025.1572494 (PMC12095336; doi:10.3389/fpls.2025.1572494)
Supplement: Supplementary file 1 [file DataSheet1.docx]

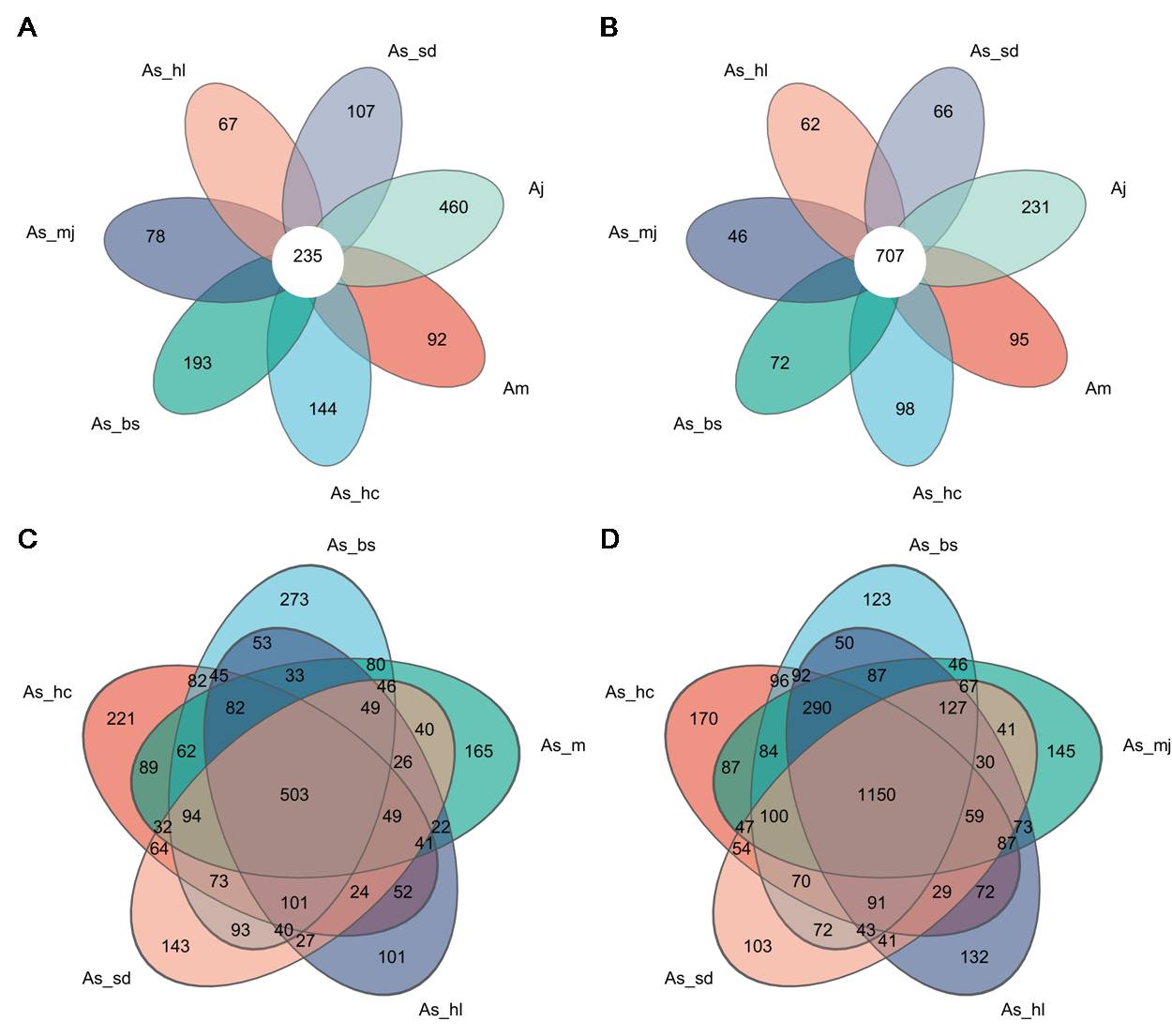


Figure S1. Venn analysis of different groups at OTU level. (A, C) Venn analysis of root nodules. (B, D) Venn analysis of soil samples. Am represent the *A. mandshurica*, Aj represent the *A. japonica*, the As_hc, As_bs, As_mj, As_hl, and As_sd represent the *A. sibirica* from five different eco-geographical environments, respectively. The same applies below.


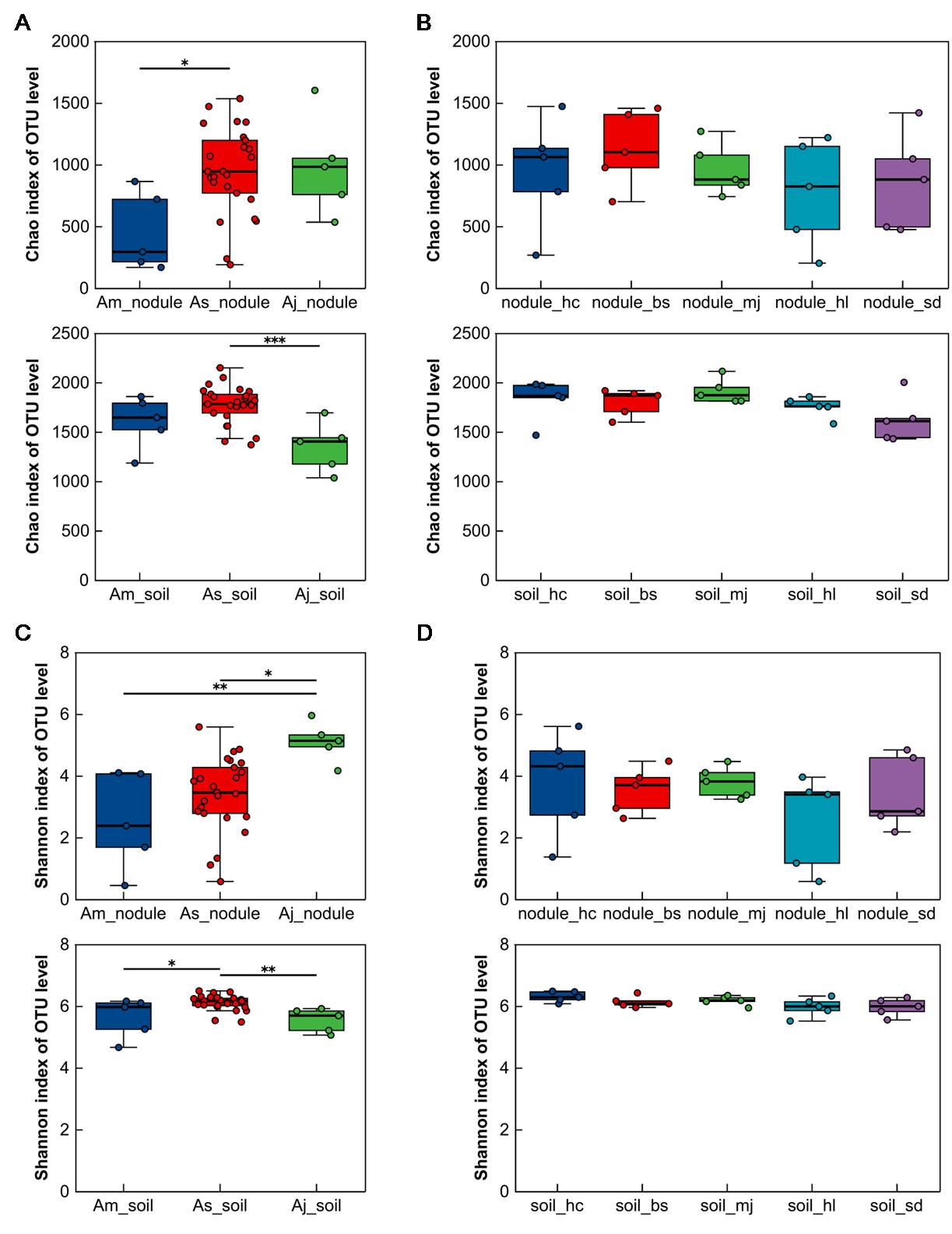


Figure S2. Microbial community α-diversity in root nodules and associated soils of three different alder species. (A) Comparative analysis of microbial community Chao index in root nodules and associated soils of three different alder species. (B) Comparative analysis of microbial community Chao index in root nodules and associated soils of *A. sibirica* from five different eco-geographical environments.  (C) Comparative analysis of microbial community Shannon index in root nodules and associated soils of three different alder species. (D) Comparative analysis of microbial community Shannon index in root nodules and associated soils of *A. sibirica* from five different eco-geographical environments. The one-way analysis of variance (ANOVA) was used to compare multiple groups. Am_nodule, As_nodule, and Aj_nodule represent the root nodules of *A. mandshurica*, *A. sibirica*, *A. japonica*, and Am_soil, As_soil, and Aj_soil represent the associated soils of *A. mandshurica*, *A. sibirica*, *A. japonica*, the nodule_hc, nodule_bs, nodule_mj, nodule_hl, and nodule_sd represent the root nodules of *A. sibirica* from five different eco-geographical environments, soil_hc, soil_bs, soil_mj, soil_hl, and soil_sd represent the associated soils of *A. sibirica* from five different eco-geographical environments, respectively. Asterisks indicate significant differences among groups, **p* < 0.05, ***p* < 0.01, ****p* < 0.001.


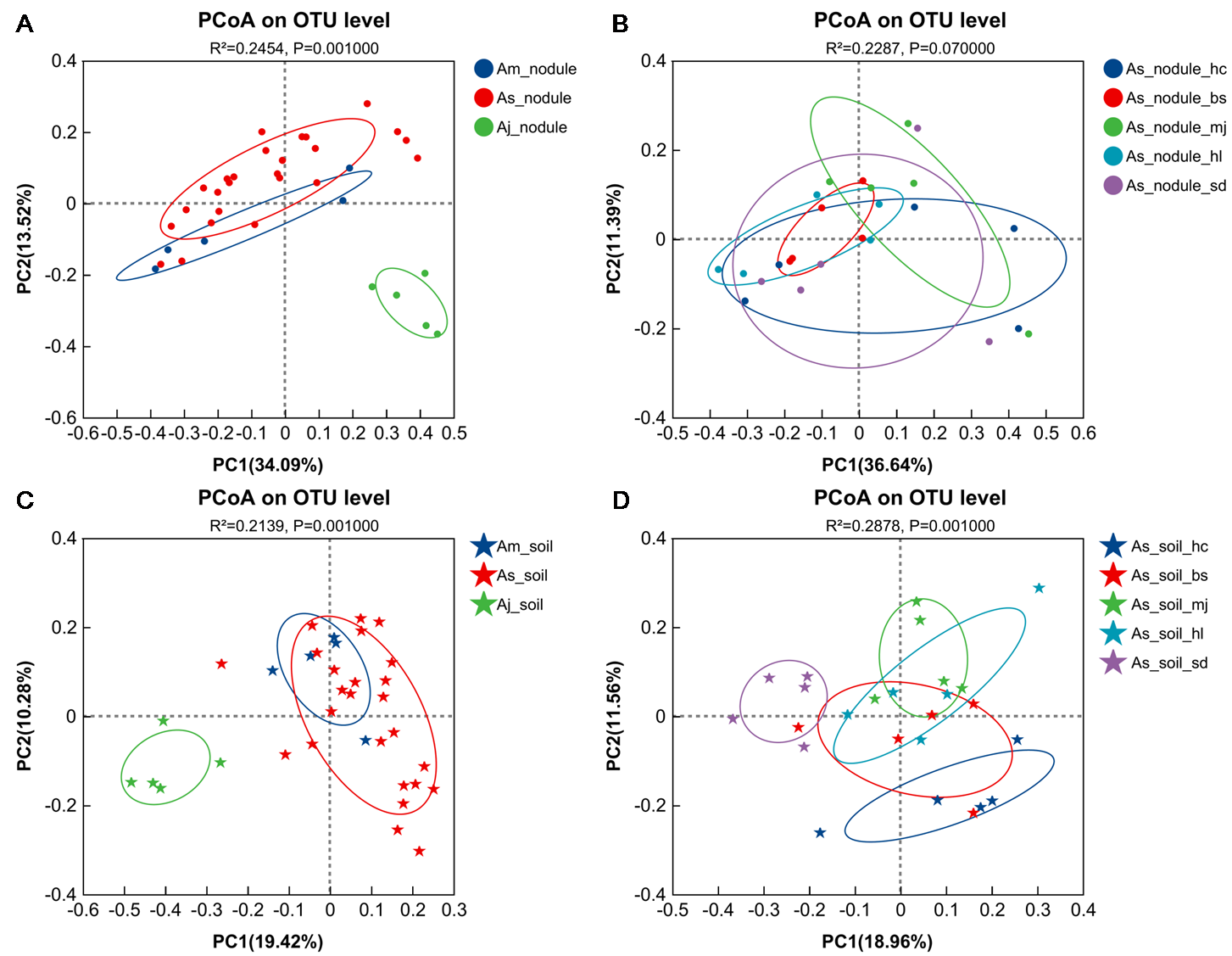


Figure S3. Microbial community β-diversity in root nodules and associated soils of three different alder species. (A) PCoA analysis of microorganisms in root nodules of three alder species. (B) PCoA analysis of microorganisms in root nodules of *A. sibirica* from five different eco-geographical environments. (C) PCoA analysis of microorganisms in associated soils of three alder species. (D) PCoA analysis of microorganisms in associated soils of *A. sibirica* from five different eco-geographical environments. PERMANOVA was used to analyze the impact of different grouping factors on sample differences. The As_nodule_hc, As_nodule_bs, As_nodule_mj, As_nodule_hl, and As_nodule_sd represent the root nodules of *A. sibirica* from five different eco-geographical environments, As_soil_hc, As_soil_bs, As_soil_mj, As_soil_hl, and As_soil_sd represent the associated soils of *A. sibirica* from five different eco-geographical environments, respectively.


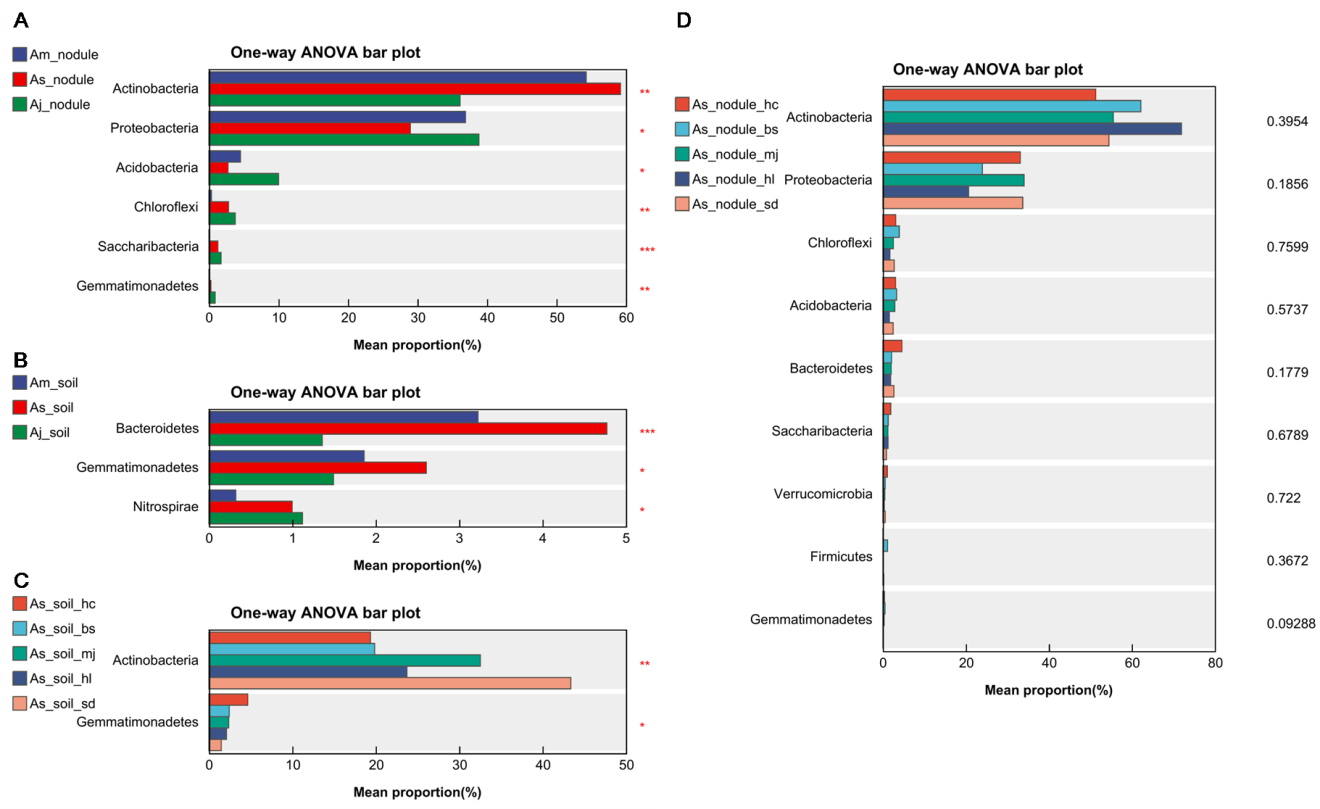


Figure S4. Difference analysis of the abundance of ten dominant phyla among different nodule and soil samples. (A) The differential abundance of dominant phyla in different root nodules. (B) The differential abundance of dominant phyla in different associated soils. (C) The differential abundance of dominant phyla in associated soils of *A. sibirica* from five different eco-geographical environments. (D) The differential abundance of dominant phyla in nodules of *A. sibirica* from five different eco-geographical environments. Only differentially abundant phyla were shown in figures A, B, and C. The one-way ANOVA was used to compare multiple groups. Asterisks indicate significant differences among groups, **p* < 0.05, ***p* < 0.01, ****p* < 0.001.


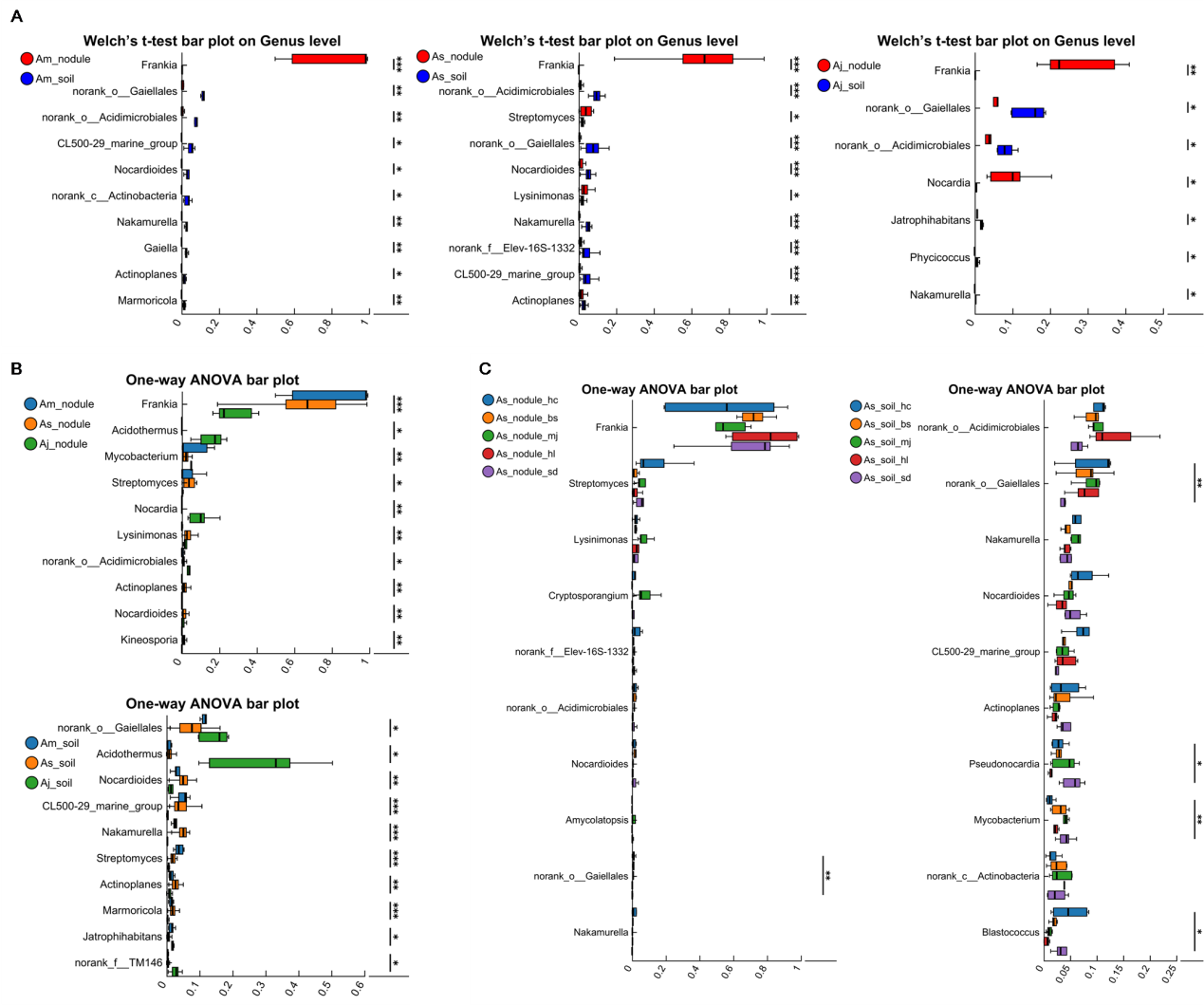


Figure S5. Analysis of abundance difference of ten dominant genera in Actinobacteria among different nodules and soil samples. (A) The differential abundance of dominant genera in Actinobacteria between the nodules and soil samples of three alder. (B) The differential abundance of dominant genera in Actinobacteria among nodules and soils of *A. sibirica* from five different eco-geographical environments. Only differentially abundant genera were shown in figures A, and B. Show the top ten dominant genera in figures C. The Welch T test and one-way ANOVA were used to compare different groups at genus level. Asterisks indicate significant differences among groups, **p* < 0.05, ***p* < 0.01, ****p* < 0.001.


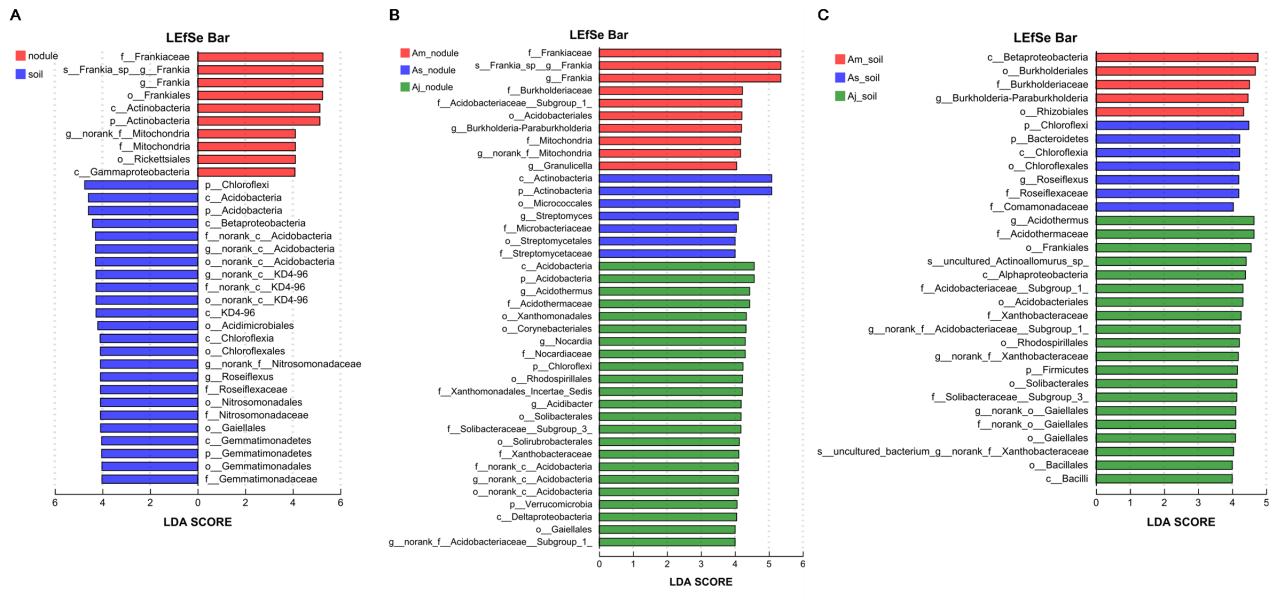


Figure S6. LDA histograms indicating the microbial communities of the alder nodules and associated soils (LDA score > 4.0, *p* < 0.05). (A) Histogram of differentially abundant genera between nodules and soils. (B) Histogram of differentially abundant genera among nodules of different alders. (C) Histogram of differentially abundant genera among soils of diverse alders. A longer bar indicates a more significant difference. The bars were colored according to the group.


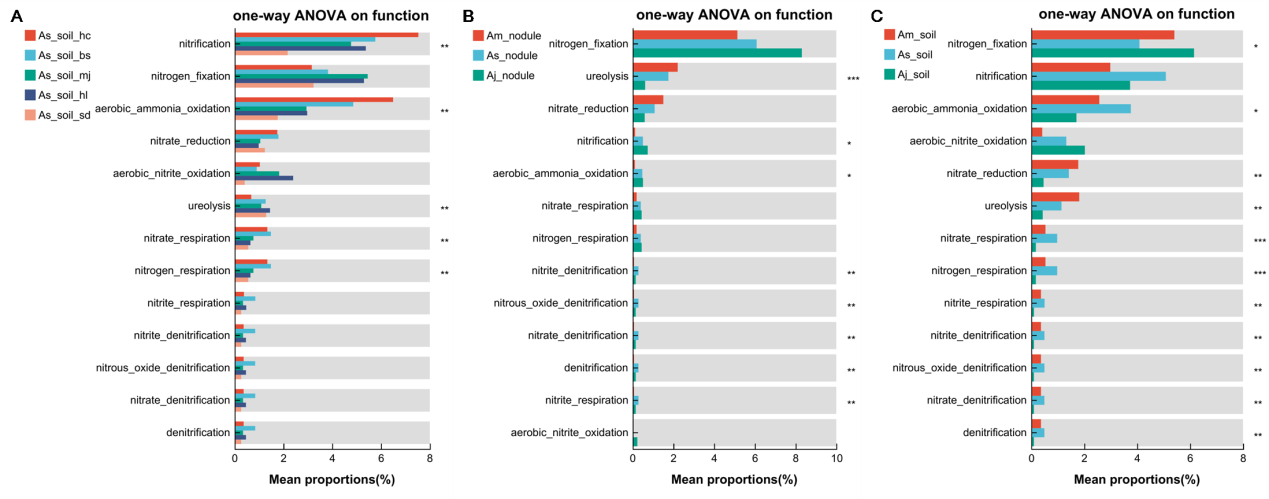


Figure S7. Comparison analysis of the abundance of the microbial nitrogen cycling pathways among different nodule and soil samples. (A) The differential abundance of the microbial nitrogen cycling pathways in associated soils of *A. sibirica* from five different eco-geographical environments. (B) The differential abundance of the microbial nitrogen cycling pathways among nodules of three alders. (C) The differential abundance of the microbial nitrogen cycling pathways among soils of three alders. The Welch T test and one-way ANOVA were used to compare multiple groups. Asterisks indicate significant differences among groups, **p* < 0.05, ***p* < 0.01, ****p* < 0.001.
